# An Immersive Virtual Reality Intervention for Preoperative Anxiety and Distress Among Adults Undergoing Oncological Surgery: Protocol for a 3-Phase Development and Feasibility Trial

**DOI:** 10.2196/55692

**Published:** 2024-05-14

**Authors:** Renée El-Gabalawy, Jordana L Sommer, Pamela Hebbard, Kristin Reynolds, Gabrielle S Logan, Michael S D Smith, Thomas C Mutter, W Alan Mutch, Natalie Mota, Catherine Proulx, Vincent Gagnon Shaigetz, Jessica L Maples-Keller, Rakesh C Arora, David Perrin, Jada Benedictson, Eric Jacobsohn

**Affiliations:** 1 Department of Anesthesiology, Perioperative and Pain Medicine University of Manitoba Winnipeg, MB Canada; 2 Department of Clinical Health Psychology University of Manitoba Winnipeg, MB Canada; 3 Department of Psychology University of Manitoba Winnipeg, MB Canada; 4 CancerCare Manitoba Winnipeg, MB Canada; 5 Department of Psychiatry University of Manitoba Winnipeg, MB Canada; 6 Harrington Heart and Vascular Institute University Hospitals Cleveland, OH United States; 7 National Research Council of Canada Winnipeg, MB Canada; 8 Department of Psychiatry and Behavioral Sciences Emory University School of Medicine Atlanta, GA United States; 9 Department of Surgery, Section of Orthopedic Surgery University of Manitoba Winnipeg, MB Canada

**Keywords:** virtual reality, preoperative anxiety and distress, perioperative mental health, breast cancer, oncological surgery

## Abstract

**Background:**

Preoperative state anxiety (PSA) is distress and anxiety directly associated with perioperative events. PSA is associated with negative postoperative outcomes such as longer hospital length of stay, increased pain and opioid use, and higher rates of rehospitalization. Psychological prehabilitation, such as education, exposure to hospital environments, and relaxation strategies, has been shown to mitigate PSA; however, there are limited skilled personnel to deliver such interventions in clinical practice. Immersive virtual reality (VR) has the potential for greater accessibility and enhanced integration into an immersive and interactive experience. VR is rarely used in the preoperative setting, but similar forms of stress inoculation training involving exposure to stressful events have improved psychological preparation in contexts such as military deployment.

**Objective:**

This study seeks to develop and investigate a targeted PSA intervention in patients undergoing oncological surgery using a single preoperative VR exposure. The primary objectives are to (1) develop a novel VR program for patients undergoing oncological surgery with general anesthesia; (2) assess the feasibility, including acceptability, of a single exposure to this intervention; (3) assess the feasibility, including acceptability, of outcome measures of PSA; and (4) use these results to refine the VR content and outcome measures for a larger trial. A secondary objective is to preliminarily assess the clinical utility of the intervention for PSA.

**Methods:**

This study comprises 3 phases. Phase 1 (completed) involved the development of a VR prototype targeting PSA, using multidisciplinary iterative input. Phase 2 (data collection completed) involves examining the feasibility aspects of the VR intervention. This randomized feasibility trial involves assessing the novel VR preoperative intervention compared to a VR control (ie, nature trek) condition and a treatment-as-usual group among patients undergoing breast cancer surgery. Phase 3 will involve refining the prototype based on feasibility findings and input from people with lived experience for a future clinical trial, using focus groups with participants from phase 2.

**Results:**

This study was funded in March 2019. Phase 1 was completed in April 2020. Phase 2 data collection was completed in January 2024 and data analysis is ongoing. Focus groups were completed in February 2024. Both the feasibility study and focus groups will contribute to further refinement of the initial VR prototype (phase 3), with the final simulation to be completed by mid-2024.

**Conclusions:**

The findings from this work will contribute to the limited body of research examining feasible and broadly accessible interventions for PSA. Knowledge gained from this research will contribute to the final development of a novel VR intervention to be tested in a large population of patients with cancer before surgery in a randomized clinical trial.

**Trial Registration:**

ClinicalTrials.gov NCT04544618; https://www.clinicaltrials.gov/study/NCT04544618

**International Registered Report Identifier (IRRID):**

DERR1-10.2196/55692

## Introduction

### Background

#### Overview

There is increasing recognition of the detrimental effects of anxiety and distress in medical populations. The National Comprehensive Cancer Network (NCCN) has advocated for psychological distress to be the sixth vital sign [1]. Both the aging demographic and overall population are increasing in Canada, which confers an elevation in the overall cases of cancer and those requiring surgery [2]. The patients’ lived experience of anxiety and distress before surgery is a near-universal and often overlooked aspect of the perioperative journey. In a study of >15,000 patients undergoing nonobstetric surgery in the United Kingdom, anxiety was rated by patients as the *worst* aspect of the perioperative experience [3]. Preoperative anxiety and distress have been shown to significantly affect negative perioperative outcomes (eg, increased hospital length of stay, pain, opioid use, and rehospitalization) [4]. However, few feasible preoperative interventions exist to mitigate preoperative anxiety and distress (hereinafter termed preoperative state anxiety [PSA]) [5].

#### PSA Risk and Defining Features

Rates of psychiatric disorders (eg, anxiety disorders and depression) are elevated across surgical samples, with particularly high rates among patients undergoing breast cancer surgery [6]. These psychiatric disorders are associated with a range of poor postoperative health outcomes [7], including increased mortality [8,9]. The presence of a psychiatric disorder at the preoperative stage is also associated with significantly greater health care costs incurred among patients undergoing breast cancer surgery [6]. A history of psychiatric disorders also increases the risk of acute PSA in elective surgery [10], although PSA can also occur outside the context of threshold psychiatric disorders. PSA is defined by anticipatory distress or anxiety related specifically to perioperative factors such as pain, loss of independence, the surgery itself, anesthesia, and death [11,12], but it can also relate to unfamiliar environments, such as the operating room (OR) and encounters with health professionals (G Klar, unpublished data, February 2024) [13]. PSA tends to be transient in nature based on a current stressor (eg, upcoming surgery); however, it can be clinically significant and debilitating in approximately 40% of patients undergoing surgery [14]. PSA is often impacted by unfamiliarity and uncertainty regarding the surgical process [15]. Research by our group and others (G Klar, unpublished data, February 2024) [12] demonstrates that PSA relates to several OR environmental stimuli, including exposure to, and placement of, the anesthetic face mask; intravenous cannula insertion; limb restraint application; and inadequate information about the intraoperative process across surgical groups. Patients undergoing oncological surgery experience elevated rates of preoperative distress, ranging from 23% to 77% in recent research [16-18]. PSA in this population has been found to relate to uncertainties of what the surgeon might find and operative procedures or regarding the effects of surgery itself [10]. The experiences with PSA of patients undergoing oncological surgery have been associated with increased postoperative pain, nausea, discomfort, fatigue, and analgesic consumption (G Klar, unpublished data, February 2024) [10-13], highlighting the significance of PSA and raising the question of whether reducing PSA can impact postoperative outcomes.

#### Interventions Targeting PSA

It is well understood that psychological states and disorders are highly responsive to a range of empirically supported targeted psychological and behavioral treatments. In light of this, there is a growing body of literature examining preoperative psychological interventions to reduce negative postoperative sequelae, with some promising but mixed results. A Cochrane review by Powell et al [19] examined the evidence on psychological preparation before surgery using general anesthesia on a range of postoperative outcomes. The authors concluded that there were significant positive effects for a range of interventions, but overall, the quality of evidence was low, which in part related to the heterogeneity of the data and the methodologies used. The interventions with the most empirical support across a range of outcomes used relaxation strategies at the preoperative stage. In oncological surgeries specifically, systematic reviews have demonstrated that preoperative psychological interventions are associated with improved outcomes, particularly patient-reported outcomes such as mood and anxiety, quality of life, fatigue, and somatic symptoms [20,21]. However, many psychological interventions, such as cognitive behavioral therapy, are high-resource approaches requiring expert administration and are not feasible to be implemented for the large and growing number of surgical interventions. This is particularly true in publicly funded and often (already) overwhelmed health care systems. Although digital technology options have addressed some of these challenges (eg, cost and accessibility), these tend to be focused on more general cognitive behavioral therapy. Formal mobile-based cognitive behavioral therapy interventions still require greater and longer patient engagement and often mental health professional support [22]. In addition, cognitive behavioral therapy is most often used to address trait anxiety or mental disorder symptomatology by dealing with maladaptive thoughts and behaviors over time [23], rather than the more transient nature of PSA, which is a psychological state directly related to the stressor [24]. Thus, virtual reality (VR) may be optimally suited to address PSA because it requires less time commitment and may be undergone at any point in the weeks leading up to surgery, providing greater flexibility. The VR technology can reduce the need for direct health care provider support and has the potential for at-home use.

The vast majority of existing studies examining nonbehavioral interventions for PSA have focused on preoperative education with mixed results and often smaller effect sizes [25]. Educational interventions typically take the form of patient reading material (eg, informational brochures), which are often underused, lack important information, are provider directed rather than patient directed, and require a higher level of health literacy [25]. Successful educational initiatives for mitigating PSA, which also translate to decreased perioperative pain and increased functioning, often include poorly feasible, high-resource initiatives such as preoperative classes led by anesthesiologists and surgeons [26]. Researchers have also examined the impact on PSA levels by enabling patients to tour the OR before surgery [27] because the OR environment is an anxiety-inducing trigger for patients undergoing surgery [12,13,28]. Although this approach has been associated with reductions in PSA levels [27], it has limited feasibility to be administered broadly due to the infrequent availability of ORs and limited resources and personnel to implement this intervention. The feasibility of such an approach is even further reduced in the context of COVID-19 and future pandemics, where hospital accessibility is restricted and maintaining sterility in patient areas such as the OR is of the utmost importance.

With respect to the use of medications for PSA, the use of anxiolytics at the preoperative stage is a somewhat controversial practice. A survey was conducted among 3661 anesthesiologists from the American Society of Anesthesiologists to determine whether anesthesiologists ask their adult patients about preoperative anxiety and what methods they use to reduce it [29]. They found that >60% of the anesthesiologists reported asking their patients about anxiety and that 91.6% of these anesthesiologists prescribed anxiety medication (most commonly, the benzodiazepine Midazolam) [29]. In the preoperative environment, benzodiazepines may be used for relaxation, sedation, ease of administering anesthesia, or the suppression of seizure activity. Although there are reasons for the use of benzodiazepines outside of PSA, they are not without risk and not ideal in all populations. Specifically, benzodiazepines can be associated with adverse outcomes such as impaired motor and cognitive functioning, delirium, and respiratory depression [30]. Benzodiazepines are also not recommended for use in older adults aged >65 years because they are at increased risk for cognitive impairment and delirium [30]. Furthermore, the use of benzodiazepines for anxiety reduction in the acute preoperative period is not associated with any differences in patient satisfaction as assessed on the day after surgery, suggesting the lack of clinical benefit for patients [31]. Other anxiolytics, such as selective serotonin reuptake inhibitors, are not suitable for PSA and are more commonly prescribed for chronic mental disorders. Pharmacological interventions also do not target the cause of anxiety experienced by patients before surgery, such as a lack of perioperative information and fear of the OR environment, nor do they provide foundational exposure (ie, exposing patients to a feared environment to reduce avoidance and promote habituation) and relaxation paradigms proven to be effective for PSA in other contexts.

#### VR Interventions

VR is a computer-generated 3D environment in which a user may be immersed through visualization and interactions. VR can be used to approximate reality to achieve ecological validity for a targeted environment and can yield affective responses [32]. VR represents a potentially novel targeted modality for PSA because it enables the integration of several effective intervention techniques, such as exposure to hospital environments, education, and relaxation strategies. VR interventions have been shown to significantly reduce anxiety and other psychiatric symptoms in other contexts (eg, fear of flying, injection phobia, and fear of heights) and are used as a predeployment prehabilitative intervention to prepare military personnel for anticipated exposure to acute stress during combat (ie, stress inoculation training [33]). Evidence suggests that prehabilitative VR interventions may be associated with reductions in psychological distress, both before [34,35] and after [36] deployment. Recently, researchers evaluated whether preoperative VR simulations that expose patients to the OR can mitigate PSA [37,38]. Preliminary research in pediatric populations has found that preoperative exposure in a VR simulation to the OR environment reduces PSA and increases compliance during the induction of anesthesia [38], and it is regarded positively and accepted by patients [39]. In recent years, some targeted VR interventions have also been developed for adult populations. Systematic reviews by Yu et al [40] and Mbewe and Smith [41] identified 5 previous studies using VR to reduce perioperative anxiety in adult patients. Yu et al [40] included studies examining both perioperative and periprocedural anxiety, and the eligibility criterion was limited to VR interventions focused on OR tours specifically. The interventions were all in video format, with the VR experiences ranging from first- [42] to third-person points of view [37,43], some of which also provided 360° visuals [44,45]. Each intervention was meant to educate the participant on the surgical procedural process and expose them to the OR environment. Results varied, with some studies reporting no differences in anxiety levels between the VR intervention and control groups, [43,44] while others found anxiety to be significantly decreased with the VR intervention [37,42,45]. Importantly, VR interventions were also found to improve patients’ procedural understanding and increase patient satisfaction and preparedness [37,45]. Mbewe and Smith [41] included 3 additional studies in their meta-analysis because they were focused on surgical cases and did not restrict VR interventions to OR tours, thus including other VR interventions (eg, nature scenes). The alternative VR interventions included a training video focused on educating patients on wide-ranging aspects of the surgical process [46], a 360° nature video with relaxing music and birdsong [47], and an audio narration of a progressive muscle relaxation technique showing a beach scene [48]. The results of the meta-analysis demonstrated that VR interventions have greater positive impacts on preoperative anxiety compared to the standard of care; however, the effect size was relatively small [41].

The impact of these VR interventions may have been limited because they lacked relevant perioperative information or exposure and only displayed re-enactments or simply exposed patients to features of the surgical process as opposed to an immersive patient-directed VR experience. Immersive patient-directed VR refers to environmental fluidity changes with head movement, which facilitates presence and immersion and deepens engagement [49]. Displaying videos offers a limited sense of presence and immersion because the user cannot directly interact with the environment, and therefore ecological validity is reduced [50]. The importance of immersion and interactivity on agency and embodied learning has been supported in prior research [51]; for example, when using VR exposure to treat anxiety disorders, first-person body experiences and other features that enhance immersion, such as movement tracking and audio and tactile exposure, have been found to be important components of effective VR interventions [52]. A sense of immersion is not only positively related to feelings of anxiety (which is a necessary component of exposure-based paradigms), but it may also enhance the effectiveness of the intervention in facilitating behavior change [52,53]. In addition, the immersive aspect of VR is biologically supported with studies demonstrating that immersing patients in a stressful VR environment can alter biomarkers of stress [54]. This initial research is promising and provides preliminary support for the utility of immersive VR to mitigate PSA, which ultimately may improve perioperative outcomes. However, one way in which this body of literature is limited is that very few, if any, studies have examined the feasibility of this type of intervention, which is a critical step given its novelty in the perioperative setting.

### Study Overview and Objectives

**Figure 1:**
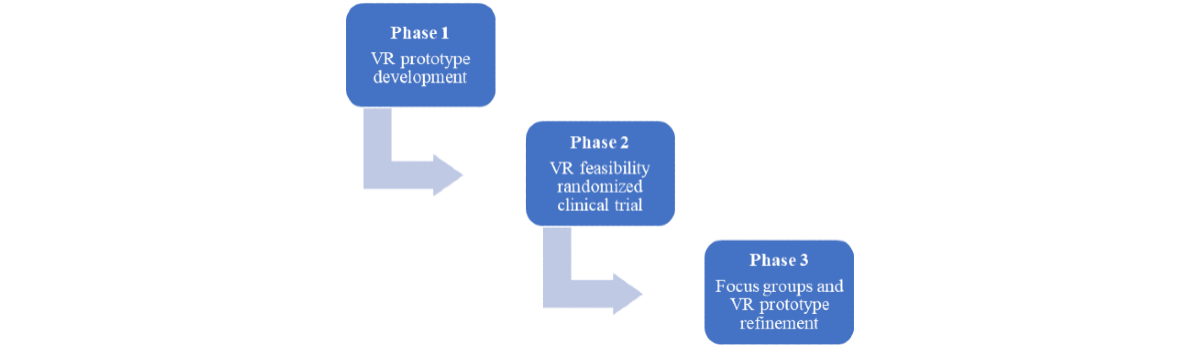
Flowchart of the 3-phase development. VR: virtual reality.

On the basis of these gaps in the literature and the importance of developing practically implemented, low-resource interventions for PSA, we designed and are evaluating the feasibility of a novel immersive VR intervention, first targeting PSA among patients undergoing breast cancer surgery. Patients undergoing cancer surgery were identified as the first target group given their high rates of PSA [16-18] and the large volume of cases (nearly 50% of patients with cancer undergo surgery), which are scheduled as elective surgeries. The use of VR is particularly advantageous for elective procedures, given that it provides the opportunity for preoperative interventions in the weeks before surgery. The development of the intervention is an iterative and collaborative process using multidisciplinary health professional input and later using focus groups of patients with lived experience. The use of both qualitative and quantitative methods promotes our ability to hear the patient voice and to make relevant adaptations to the application as directed by our analysis based on the feasibility and focus group findings. This will be achieved in 3 phases as depicted in Figure 1, developed in line with international guidelines established by the Virtual Reality Clinical Outcomes Research Experts (VR-CORE) committee for VR trials in health care [55]. Phase 1 (completed; in line with the framework from the VR-CORE guideline’s VR1 phase) involved the development of an initial VR prototype that was developed in conjunction with health care professionals working in the OR and engineers (authors MSDS, CP, and VGS) at the National Research Council of Canada [56]. This prototype comprises an immersive VR representation of the OR and patient induction process for an individual undergoing breast cancer surgery with general anesthesia. Phase 2 (underway; in line with the framework from the VR-CORE guideline’s VR2 phase) uses a randomized design (VR intervention vs VR control [ie, Nature Treks VR] vs treatment as usual [TAU]) to assess feasibility on a sample of patients undergoing breast cancer surgery. The feasibility study used quantitative scales to understand the recruitment and randomization capability, acceptability, and feasibility of the study design, along with preliminary utility for reducing PSA. In phase 3 (underway; also in line with the VR-CORE guideline’s framework VR1 phase because we are collecting user-testing feedback to improve the VR content), we are conducting focus groups with select participants from the feasibility trial, and the results based on the focus groups, along with the feasibility findings, will inform continued refinements of the prototype for a future randomized clinical trial (in line with the framework from the VR-CORE guideline’s VR3 phase). An effective PSA-reducing intervention has the potential to mitigate poor perioperative mental and physical health outcomes in patients undergoing oncological surgery and yield significant cost savings to the overall health care system. An initial successful VR platform could be modified and applied to other surgical populations in the future.

## Methods

### Phase 1 (Complete)

#### VR Development

Phase 1 involved the development of the initial VR prototype, developed in the Unity game engine (Unity Technologies) and deployed using the Oculus Rift S headset (Meta). We aimed to develop the initial VR prototype to simulate an OR and anesthetic induction at the Health Sciences Centre (HSC) in Winnipeg, Manitoba, Canada, a tertiary care university-affiliated hospital connected to a provincially mandated cancer agency. With institutional approval, several photographs and videos were taken of the OR, and 1 live induction process was audio and video recorded (refer to the *Ethical Considerations* section for consent details). Furthermore, an email was sent to all attending anesthesiologists at the HSC requesting them to provide their standard patient *induction and safety scripts*. We received 4 scripts that were compared, and consistent elements across the scripts were amalgamated into a *standard script* to be integrated into the VR simulation.

**Figure 2:**
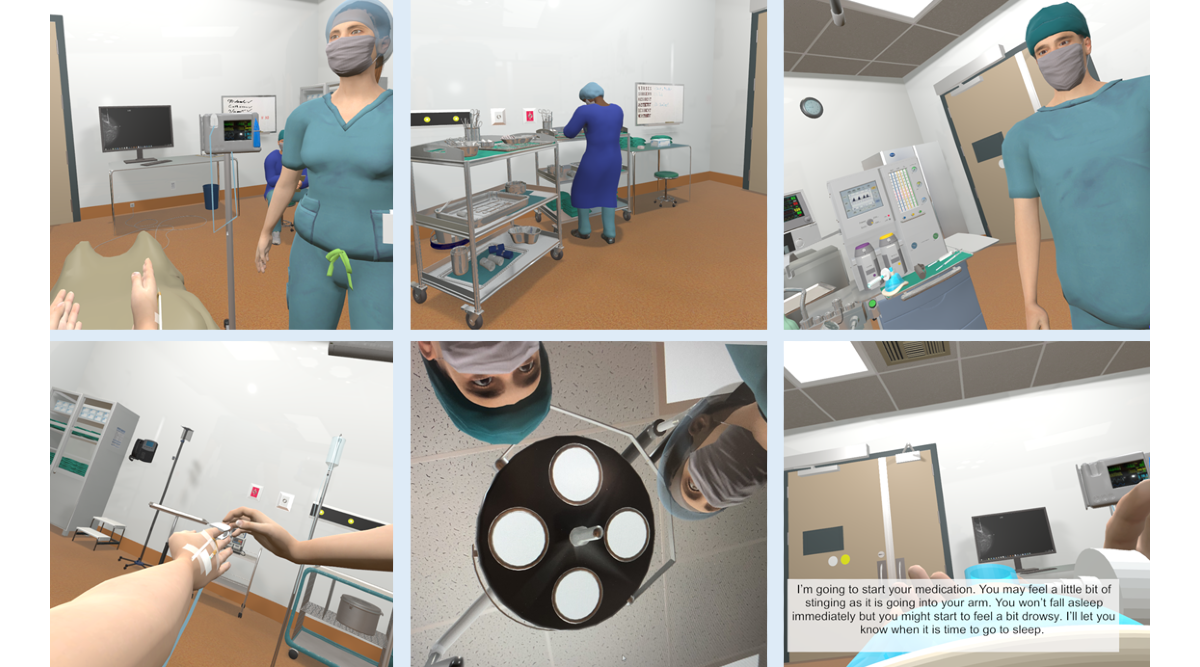
Screenshots of the immersive virtual reality operating room environment.

**Figure 3:**
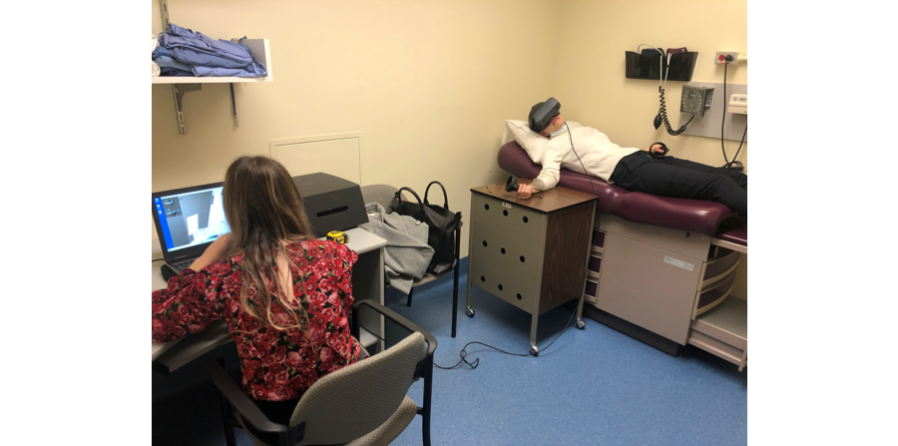
Virtual reality administration and setup.

An iterative and collaborative development process was used to create the VR environment and simulation. The initial prototype was based on all elements included in an induction sequence on a real patient, which was filmed. On the basis of input and feedback from coauthors and OR professionals at the HSC in addition to consultations between coauthors and the VR development team at the National Research Council, further elements to be implemented (equipment, personnel, narrations, animations, etc) were identified and prototypes were developed. In the interest of having a prototype to test in a timely manner and to reserve the bulk of the grant funds to commit to the development of the intervention after patient feedback via the feasibility trial and focus groups, specific decisions were made to animate aspects in the OR that have been reported by patients to be the most anxiety provoking (G Klar, unpublished data, February 2024). Prototypes were trialed regularly by coauthors and feedback incorporated in the next round of refinement. Phase 1 commenced in November 2019 and was completed by April 2020 (refer to Figure 2 for screenshots of the immersive VR OR environment and Figure 3 for the administration setup). A manuscript detailing the technical aspects of the VR creation is in development.

#### Components of the Initial VR Prototype

The user starts the simulation seated on a virtual operating table in the OR. The user is provided a minimum duration of 5 minutes to visually *explore* the environment, which includes surgical (eg, instruments, lights, medical devices, sterile items, and x-ray images) and anesthetic (eg, medications and anesthetic monitors) items. The environment also includes background noises (eg, a beeping monitor) and lighting consistent with the OR environment. Several health care personnel (ie, an anesthetist, a surgeon, and 2 nurses), including a woman and a racial and ethnic minority person, are present in the room. After at least 5 minutes of exploration, an induction narrative simulation commences. The patient is instructed to lie supine (on the virtual OR table) and is taken through a mock anesthetic induction process from a first-person perspective. For the initial prototype, the patient’s avatar was developed as White and gender neutral. One of the virtual nurses and the virtual anesthetist speak to the patient and walk them through the steps while the procedures are being performed. The narrative component includes describing surgical procedures such as attaching electrocardiogram stickers, attaching a pulse oximeter, completing a safety briefing, and placing an oxygen mask over the patient’s face. The additional steps of attaching a blood pressure cuff, attaching an intravenous line to the cannula on the hand, and injecting antibiotics and anesthesia are described but not animated in the VR. As mentioned previously, the animation of these steps was not included due to time and funding constraints, along with the desire to have the final simulation (including the animated components) be patient informed. At the point in the virtual induction where the patient would be falling asleep, the VR screen fades to black, and the simulation ends. To further encourage immersion, the induction script includes periods of patient engagement, including having the patient place their right arm correctly for the nurse and specifying their name, date of birth, type of surgery, and whether they have any allergies (as would be done as part of the mandatory World Health Organization surgical safety briefing). All this is done in real time. The OR induction process is controlled by a combination of autoadvancement methods, based on the detection of certain physical positioning requirements, and input from research personnel who progress the patient through each phase of information delivery and patient engagement (eg, responding to questions). However, haptic feedback to participants was not provided because this was beyond the scope of the study objectives, and the resources available at the time were insufficient.

### Phase 2 (Completed)

#### Study Design

This study used a single-center feasibility and pilot randomized clinical trial with 3 arms. The study was registered on ClinicalTrials.gov (NCT04544618) on September 10, 2020 [57].

#### Overview

Phase 2 assessed the feasibility and preliminary outcomes of the VR OR prototype among patients undergoing breast cancer surgery. Specifically, with respect to the feasibility of this intervention, this study evaluated (1) recruitment capability and the characteristics of the resulting sample, (2) data collection procedures and selection of an appropriate outcome measure of PSA, and (3) participant acceptability and suitability of the VR OR intervention (ie, the active intervention) as well as the inclusion and acceptability of a VR control group (Nature Treks VR; ie, a non-OR immersive VR nature environment) and a TAU group. In addition, as a final aim, this study (4) pilot-tests the preliminary impact of the active intervention on PSA.

This study aimed to recruit 45 participants (n=15, 33% randomized to each arm [ie, active intervention, VR control, and TAU]). This target sample size is consistent with recommended guidelines for determining the sample size of a feasibility study and previous studies of this nature [58,59]. Furthermore, as an aim of this feasibility study, we are evaluating the extent to which we are able to recruit participants within the target population, which will inform modifications to the recruitment method as well as the expected duration of the upcoming randomized controlled trial (RCT).

#### Inclusion and Exclusion Criteria

The inclusion criteria for participation are as follows: (1) being aged ≥18 years, (2) able to speak and read English, (3) having a breast cancer diagnosis, and (4) scheduled or being scheduled to undergo breast cancer surgery under general anesthesia at the HSC. Those who do not meet these criteria, are not competent to provide informed consent (eg, due to cognitive impairment), or are unable to participate in a VR intervention (eg, due to significant visual or auditory impairments) are excluded.

#### Recruitment

Patients undergoing oncological surgery are recruited from the HSC via posters, patient surgical oncology appointment, or preoperative education class at the Shared Health Breast Health Centre (ie, a public health breast center that coordinates clinical assessment, diagnostic tests, treatment, education, and support) or the Breast & Gyne Cancer Centre of Hope (ie, resource center). A staff person or physician at the Breast Health Centre or Breast & Gyne Cancer Center of Hope (or research coordinator) briefly describes the study, and the contact information of interested patients is recorded. Recruitment posters with study staff’s contact information are posted at the Breast Health Centre, Breast & Gyne Cancer Center of Hope, the HSC, and on the web for any potential participants not identified at the time of their surgical oncology appointment or the preoperative education class.

#### Protocol

Phase 2 used a single-blind randomized design (1:1:1 randomization; stratified according to surgery type [with vs without reconstruction] and whether neoadjuvant chemotherapy was received; stratification enables equal proportions of participants with these characteristics across each of the 3 study groups), using quantitative and qualitative methodologies. This was done to assess the feasibility of, and pilot-test, the VR simulation to expose patients undergoing breast cancer surgery to the OR and induction process (ie, VR OR) compared to a non–surgery-related VR simulation (ie, VR control) and TAU. All participants complete self-report measures approximately 2 weeks before surgery (ie, baseline; for VR groups, this will occur on the day of the VR visit as well as before and after testing the intervention), on the day of surgery (preoperative period), 5 days after surgery (acute postoperative period), and 30 days after surgery (30-day postoperative follow-up).

Randomization is carried out using a web-based random number generator (details of how these random numbers are generated are available on the website) [60]. A master file was created in Excel (Microsoft Corp) by author JS, which consisted of 4 different stratification groups to correspond to the number of possible combinations: no chemotherapy and no reconstruction; no chemotherapy, with reconstruction; chemotherapy, no reconstruction; and chemotherapy and reconstruction. Using the web-based number generator, each stratification group was populated with equal proportions of the randomly ordered numbers (1, 2, or 3) that corresponded to the intervention groups to which a participant could be assigned (1: OR intervention; 2: Nature Treks VR intervention, and 3: TAU). Only research personnel who randomized the participants had access to the file. Due to the nature of this study, only partial blinding was possible. Specifically, those in the TAU group received no VR intervention, but those randomized to VR control versus the VR active intervention were blinded. Participants randomized to either the active intervention or VR control group schedule a meeting date to undergo the VR intervention, approximately 2 weeks before their surgery, during their telephone call with the study coordinator. On the day of the intervention meeting, participants complete baseline questionnaires before the intervention and additional questionnaires after the intervention (detailed in the *Measures* section). Those randomized to TAU are either emailed a link to complete the baseline measures using Qualtrics software (Qualtrics International Inc) or are mailed a hard copy, depending on their preference, 2 weeks before their surgery (baseline). On the day of surgery (preoperative period), participants again complete the measures assessing PSA that had been assessed at baseline. Five days after surgery (acute postoperative period), participants will complete the PSA measures, in addition to the other initial baseline measures. Finally, all baseline measures are readministered at the 30-day postoperative follow-up. For both postoperative follow-ups, participants have the option to receive hard copies of the measures via mail (to complete within 72 hours) or a survey link to complete the measures on the web.

For the active intervention group, the VR simulation begins with the patient sitting on a hospital bed, wearing the VR headset, and holding the controllers. The details of the final prototype are described in the *Phase 1 (Complete)* section and represented in Figure 2. For the exploration component, the patient is instructed to explore the VR OR for a minimum of 5 minutes, although the exploration period can last longer (the total duration engaged in the simulation is tracked for each participant). After the exploration period, the scripted portion of the simulation begins. As detailed previously, the simulation ends after the virtual oxygen mask is placed on the patient’s mouth, and the screen darkens. The scripted portion of the simulation is approximately 3 minutes long (ie, participants will spend a minimum of 8 minutes engaged in the VR intervention).

Participants randomized to the control intervention have the opportunity to explore a non–surgery-related VR simulation preprogrammed in the VR goggles (ie, Nature Treks VR experience). Participants are instructed to explore a selected nature environment for a minimum of 10 minutes, and their total duration engaged with the simulation is tracked. Participants in the TAU group receive the standard of care; they receive no additional intervention and have the option to receive information at their surgical oncology appointment and attend preoperative education classes, which all patients have the opportunity to attend. After the completion of the study, all participants are provided with a debriefing form that explains the purposes of the study.

#### Measures

A variety of self-report measures are administered to participants across 4 time points of this study (ie, baseline or VR visit, preoperative period, acute postoperative period, and 30-day postoperative follow-up). Baseline measures are administered approximately 2 weeks before surgery (at the VR intervention visit for the intervention groups or via mail or web-based for the TAU group). These measures include a background sociodemographic questionnaire (eg, assessing age, marital status, diagnosis, type of surgery, previous history of surgeries, and mental health diagnoses); the Preoperative Intrusive Thoughts Inventory (PITI) [61]; the Amsterdam Preoperative Anxiety Information Scale (APAIS) [62]; the NCCN Distress Thermometer (a visual analog scale; also adapted for anxiety) [63,64]; the Peritraumatic Distress Inventory [65]; the Primary Care Screen for Posttraumatic Stress Disorder Diagnostic and Statistical Manual of Mental Disorders, Fifth Edition [66]; the Brief Resilient Coping Scale (BRCS) [67]; and the Patient-Reported Outcomes Measurement Information System (PROMIS) anxiety [68], depression [68], global health [69], fatigue [70], and emotional support scales [71]. The sociodemographic data collected were informed by the methodology used in a previous study by Grocott et al [72] that itself informed the current methodological approach. All mental health symptom scales are empirically validated self-report measures in various health populations, languages, and cultures. During the VR simulation, we also monitor participants’ skin conductance (using eSense Skin Response [Mindfield Biosystems Ltd]) and heart rate (using a Fitbit device [Google LLC]) while engaged in the VR simulation; participants are asked to report their level of distress and anxiety (using the NCCN Distress and Anxiety Thermometer scale ranging from 0 to 10) during the intervention (approximately 7 min from the start time). Throughout the simulation, research personnel complete a standardized behavioral observation form recording any notable verbal or nonverbal indications while in the intervention. After the simulation, the intervention groups complete the Igroup Presence Questionnaire [73] and a patient acceptability questionnaire (developed by the research team and including both closed-ended and open-ended items for participants to describe their impressions of the intervention). The patient acceptability questionnaire contains questions pertaining to rating the extent (on a scale ranging from 0% to 100%) to which the participant agrees to 13 statements about the VR intervention (eg, “I found the VR intervention was helpful” and “The VR intervention worsened my anxiety/concerns about my surgery”), open-ended and closed-ended questions about motion sickness, open-ended questions about what the participant liked and disliked and what they found helpful about the VR (if applicable), open-ended and closed-ended questions about whether the VR intervention was worthwhile, and a closed-ended question about other elements that should be included in the VR OR simulation.

On the day of surgery, all participants are asked to complete the PITI, the APAIS, and the NCCN Distress and Anxiety Thermometer either while they are in the waiting room or in the preoperative holding area (depending on when is most convenient and the timings of surgeries). Furthermore, we again monitor participants’ skin conductance and heart rate at this time. Participants are also asked to provide an additional rating for the NCCN Distress or Anxiety Thermometer while in the OR; instead of using the paper copy in the OR, the study coordinator or anesthesiologist asks participants to verbally indicate their current level of distress and anxiety (ie, on a scale ranging from 0 to 10). Five days after surgery, participants are asked to complete the NCCN Distress and Anxiety Thermometers; the PROMIS anxiety, depression, global health, fatigue, and pain intensity scales; a visual analog anxiety and distress graph; a postoperative summary form (to capture length of hospital stay and impressions of the various preoperative anxiety and distress measures); the BRCS; and the Peritraumatic Distress Inventory. Those in the active intervention group also receive open-ended questions requesting feedback to inform modifications that should be made to the second model of the simulation (in phase 3). Finally, 30 days after surgery, participants are asked to complete the NCCN Distress and Anxiety Thermometers; the PROMIS anxiety, depression, global health, fatigue, and pain intensity scales; the BRCS; and the Primary Care Screen for Posttraumatic Stress Disorder Diagnostic and Statistical Manual of Mental Disorders, Fifth Edition.

As will be detailed in the feasibility manuscript, although a number of self-report and objective measurements were obtained, the primary measures relate to feasibility (eg, recruitment and dropout rate as well as VR impressions and feedback). This feasibility study will also evaluate the extent to which these measures are completed as instructed and best represent PSA and whether preliminary trends support decreases in PSA across the study duration. The primary outcome measure for the larger future clinical trial will be determined based on this study. The proposed primary outcome measures of interest include the PITI, the APAIS, and the NCCN Distress and Anxiety Thermometers.

#### Data Analysis

Quantitative data will first be analyzed descriptively: the recruitment, engagement, and attrition rates will be calculated; sample characteristics will be summarized (both across the total sample and within each group); and mean scores on measures will be reported (within each group). The assessment of acceptability will be based on open-ended qualitative feedback regarding impressions of the VR intervention, which will be analyzed using content analysis with NVivo 12 (Lumivero), which was designed to assist with qualitative data organization. We will also determine acceptability based on the developed Likert scale questions. Means or medians will be reported depending on the distribution of the data. A triangulation approach will be used to amalgamate data sources and comprehensively assess acceptability. Paired samples *t* tests (1-tailed) and repeated measures ANOVAs will assess whether there are changes in participant-reported symptoms across the duration of the study within the active intervention group. If power permits, independent samples *t* tests (or an ANOVA, followed by post hoc pairwise comparisons) will assess whether there are mean differences in PSA scores between (1) the active and control intervention groups and (2) the active intervention group and TAU (this will be examined descriptively if underpowered). We will also examine changes in physiological arousal (an objective indicator of distress), assessed via skin conductance and heart rate, between and within (ie, during VR intervention vs within OR) intervention groups, and we will assess whether self-reported distress and anxiety scores are correlated with the indices of physiological arousal. Physiological data will also be integrated in the VR technological paper (in development) to understand whether physiological indices change during the VR experience (indicative of immersion). Finally, if power permits, independent samples *t* tests and ANOVAs will assess whether there are mean differences in patient-reported symptoms according to certain sample characteristics (eg, age, clinically significant anxiety, history of previous surgery, and type of surgery), both within the active intervention group and within the complete sample.

### Phase 3 (Underway)

#### Focus Groups

The focus groups are being led by 4 authors (RE, KR, GSL, and JB). The first focus group consisted of individuals randomized to the VR intervention (ie, those who received the VR OR intervention before surgery) and took place over Zoom (Zoom Video Communications, Inc). A second focus group was then conducted with individuals randomized to the TAU group or the VR control group (ie, those who did not receive the VR intervention). This focus group took place in person at the HSC and commenced with each participant trialing the VR OR prototype. Due to scheduling conflicts, we held an additional focus group for TAU participants in December 2023, and an additional focus group with VR intervention participants in February 2024. It is important to note, as we discuss in the *Results* and the *Limitations* sections, that the VR control group (ie, Nature Treks VR experience) ended up being dropped due to slow recruitment; thus, we only ran the control focus groups with individuals randomized to TAU.

The focus groups commence by reviewing the purpose of the feasibility study and the VR prototype. A semistructured question guide was developed by the research team, including several open-ended questions regarding patients’ experiences with the VR prototype, whether they believe it impacted their surgical experience, and recommendations for further development. After open-ended questions are asked, more specific questions regarding feedback on potential adaptation of the prototype are explored. The focus groups are audio recorded and transcribed using Trint software (Trint Limited). Qualitative data will be analyzed using reflexive thematic analysis [74-76], examining themes identified by the authors across participants.

#### VR Refinement

On the basis of the results of phase 2 and the focus groups, additional modifications and changes are being made to the VR prototype. As part of the feasibility study, participants from phase 2 were asked about any additional elements to be included in the VR simulation. In addition, a coinvestigator meeting was held in April 2023 where preliminary results from a case series proof-of-concept study (first 7 participants of the feasibility trial) were presented. The expert coinvestigators provided additional input on modifications based on these initial case series findings. On the basis of the findings from the feasibility trial, expert input, and focus groups, a list of additional elements to be integrated into the VR will be developed (eg, artificial intelligence integration, educational component, guided relaxation strategies, and exposure to the waiting room or recovery environments). People engaging with the VR OR will also be provided with the opportunity to customize their avatar to be more representative of themselves. This means participants will be able to change the avatar’s skin color and height to enhance embodiment. Efforts will also be undertaken to improve the diversity of the VR OR staff.

On the basis of the results from the feasibility study, focus groups, and expert input consensus, we will aim to complete the final prototype by mid-2024 for testing in a future randomized clinical trial.

### Ethical Considerations

#### Phase 1

No patient participants were involved in the development of the initial VR prototype; therefore, ethics approval was not required. However, for the audio and video recording of a live anesthetic induction process at the HSC, we obtained signed patient consent (of note, we excluded any visual of the patient).

#### Phase 2

We received ethics approval from the University of Manitoba Health Research Ethics Board on June 30, 2020 (HS23957). Ethics amendments were approved in January 2021, September 2021, April 2022, and November 2022 (Multimedia Appendix 1). Participants first gave verbal consent to be contacted for research purposes after being briefly told about the study at the individual’s surgical oncology appointment or at a preoperative education class. Alternatively, if learning about the study from a poster, patients contact our research team directly (as described in the *Recruitment* section). Participants are randomized during the recruitment call if they provide verbal consent to participate. Furthermore, we obtain verbal consent over the telephone from participants in the screening period and written informed consent before their participation in the study (via mail or email). All data are stored deidentified. Participants received a $25 CAD honorarium for participating in the feasibility trial.

#### Phase 3

In June 2023, we received ethics approval to conduct the focus groups from the University of Manitoba Health Research Ethics Board (HS26054) to recruit participants from phase 2 who indicated on their consent form that they would be willing to serve as a patient partner or adviser for future development of the VR program. For those indicating *yes*, a research assistant followed up via telephone to assess continued interest in involvement. All focus group transcript data are deidentified. All focus group participants received a $20 CAD honorarium for participating in the focus groups.

## Results

This study was funded by the Government of Canada’s New Frontiers in Research Fund, an operational research grant program supporting high-risk and high-reward transformative research, awarded to author RE as principal investigator in March 2019. Two extensions were provided on funding related to COVID-19 delays and parental leave of the principal investigator (RE). Author JS led a component of the feasibility trial for the purposes of her doctoral dissertation and, given the novelty of this area, analyzed data from the first 7 participants in a proof-of-concept case series format [77]. Phase 1 was completed in November 2021, and recruitment for phase 2 commenced in December 2021 and was completed in December 2023. On the basis of some of the initial challenges with recruitment, such as institutionally mandated shutdowns of clinical research, there were a few amendments to the initial protocol (Multimedia Appendix 1). The 2 major changes to the protocol were initiated because of challenges with recruitment, which were related to the COVID-19 pandemic restrictions, including university-wide research shutdowns, and changes to the formats of the Breast Health Centre’s educational sessions (classes became virtual). We opted to exclude the VR control arm for the purposes of feasibility and proceeded with a 2-armed trial (ie, VR intervention vs TAU). We also expanded recruitment to all major oncological surgeries (instead of restricting it to breast cancer surgeries) in November 2022. However, as of August 2023, no patients undergoing non–breast cancer surgery have been recruited. We conducted the first 2 focus groups in August and September 2023, and the final focus group was completed in February 2024. We will submit full feasibility results and results of the focus groups for publication after the completion of data analysis in summer 2024. After the final development of the VR program, we will apply for additional funding in winter 2024 to conduct an RCT of the final VR simulation in 2025. We will develop a separate protocol outlining the details of the final prototype and subsequent RCT.

## Discussion

### Summary

Despite the high prevalence of PSA and the significant health-related impact of PSA on perioperative outcomes, little research has examined feasible and accessible interventions. Empirically supported treatments for generalized anxiety, such as cognitive behavioral therapy, are not obtainable before surgery for most patients in Canada due to limited access to mental health professionals and limited time before surgery. To the best of our knowledge, there has not been any comprehensive immersive VR simulation to be implemented before surgery. An easily accessible simulation using VR has the potential to reduce PSA for a large number of Canadians, which may yield better postoperative health outcomes as well as cost savings to the health care system as a whole. The final novel VR intervention that we are developing is intended to be able to be used by patients independently (including in the comfort of their own homes), but it could also be offered in hospital settings (including preanesthesia clinics) and be able to be easily customized for different surgical procedures under general anesthesia. The integration of VR in health care shows tremendous growth and promise [78]. Headsets are now commercially available at low cost, and advances in the technology create growing opportunities for easy implementation (eg, stand-alone wireless VR headsets). It is expected that the release of the Apple Vision Pro, the company’s first augmented and VR headset that was unveiled in June 2023, will further increase awareness, demand, and accessibility of VR.

### Limitations

The development of this research took place during the peak of the COVID-19 pandemic, and therefore, there were unanticipated recruitment challenges and overall delays in development. First, lockdown orders initially slowed the development and refinement of the VR OR prototype itself due to work-from-home orders. Once the prototype was completed, there were further issues due to surgery delays, along with advisories against *unnecessary* hospital visits, and changes to how patients were initially approached for their interest in participating (the Breast Health Centre changed the format of education classes from in-person to virtual sessions, and eventually some classes were canceled altogether). Ultimately, from December 2021 to December 2022, only 8 participants were recruited. Of note, during this time, there were also intermittent periods where no clinical research could be conducted. However, from January to September 2023, an additional 15 participants were recruited. This increase in recruitment may relate to changes in societal and health care practices (eg, vaccinations, reduced COVID-19 hospitalizations, and the removal of public health mandates) and will be explored further in the feasibility analysis.

Although feasibility and pilot studies are critical in the development of new interventions, the generalizability of the findings is limited. Therefore, when published, the results of the feasibility trial will need to be interpreted with caution. Relatedly, the focus of these 3 initial phases has been on patients undergoing oncological surgery, namely patients undergoing breast cancer surgery, which may differ in important ways from other noncancer surgical samples. In addition, within the VR program itself, there was 1 variation of the patient avatar, and it does not reflect differences in gender, body type, or ethnicity, which may potentially decrease patient embodiment within the VR intervention and the ecological validity on the day of surgery. Furthermore, we did not ask background questions related to ethnicity, sexual orientation, or disability and, therefore, are limited in understanding feasibility aspects across diverse populations. The sample size is also underpowered to reliably detect effects but will be able to inform future RCTs in terms of design and implementation. Furthermore, preliminary trends in the data will increase confidence in the potential utility of the intervention. Relatedly, the exclusion of the VR control group (ie, non-OR VR) as per our amendment limits our ability to understand preliminary effects of the VR intervention. It is possible that any anxiety-reduction trends for the VR intervention group compared to TAU may relate to a factor other than the effect of the intervention itself (eg, an additional appointment before surgery and the opportunity to discuss fears). It will be particularly important for future RCTs to include a comparable comparison group to elucidate potential mechanisms. A future RCT is planned to test the final iteration of the VR OR, and we intend to compare it to a mobile phone–based intervention using the same components of the simulation as well as TAU. This will allow us to disentangle the potential additive effect of immersion that exists only in VR applications. Finally, recent research by our group [72] and others demonstrates that PSA for oncological surgery relates to a range of factors, including uncertainty of the health trajectory, recovery [79,80], and the psychosocial implications of surgery. Our prototype intervention only targets 1 component of PSA (ie, exposure to the OR and information regarding induction), which may not result in meaningful reductions among those with PSA related to other factors. However, the results from these 3 phases will allow us to refine the VR intervention to best meet patients’ needs and integrate all possible features to further ameliorate PSA (eg, relaxation strategies). Future versions of the simulation may also include advanced VR features, such as relevant haptic feedback, as the technology develops further.

### Conclusions

We are developing, testing, and refining a novel VR intervention aimed to reduce PSA before major oncological surgery using a general anesthetic. The development of the VR simulation is guided by both experts in the field and people with lived experience. VR is a promising tool, given its ability to be broadly disseminated. This study will lay the groundwork for a promising intervention to reduce PSA before major oncological surgery, and future iterations could be easily adapted to other forms of surgery. This ultimately may have significant positive effects on patient health postoperative outcomes and patient experience, along with cost savings for health care systems in the future.

## Appendix

Multimedia Appendix 1 Virtual reality study ethics amendment submission details.

## Abbreviations

APAIS: Amsterdam Preoperative Anxiety Information Scale
BRCS: Brief Resilient Coping Scale
HSC: Health Sciences Centre
NCCN: National Comprehensive Cancer Network
OR: operating room
PITI: Preoperative Intrusive Thoughts Inventory
PROMIS: Patient-Reported Outcomes Measurement Information System
PSA: preoperative state anxiety
RCT: randomized controlled trial
TAU: treatment as usual
VR: virtual reality
VR-CORE: Virtual Reality Clinical Outcomes Research Experts

## References

[1] Holland JC, Bultz BD (2007). The NCCN guideline for distress management: a case for making distress the sixth vital sign. J Natl Compr Canc Netw.

[2] Brenner DR, Poirier A, Woods RR, Ellison LF, Billette J, Demers AA, Zhang SX, Yao C, Finley C, Fitzgerald N, Saint-Jacques N, Shack L, Turner D, Holmes E (2022). Projected estimates of cancer in Canada in 2022. CMAJ.

[3] Walker EM, Bell M, Cook T, Grocott M, Moonesinghe S, Central SNAP-1 Organisation, National Study Groups (2016). Patient reported outcome of adult perioperative anaesthesia in the United Kingdom: a cross-sectional observational study. Br J Anaesth.

[4] Abate SM, Chekol YA, Basu B (2020). Global prevalence and determinants of preoperative anxiety among surgical patients: a systematic review and meta-analysis. Int J Surg Open.

[5] Vine M, Joseph K, Gibson D, Lim B, Chua M, Siu AH, Dooreemeah D, Lee A, Cuomo R, Seth I (2024). Innovative approaches to preoperative care including feasibility, efficacy, and ethical implications: a narrative review. AME Surg J.

[6] Fox JP, Philip EJ, Gross CP, Desai RA, Killelea B, Desai MM (2013). Associations between mental health and surgical outcomes among women undergoing mastectomy for cancer. Breast J.

[7] Hanrahan NP, Bressi S, Marcus SC, Solomon P (2016). Examining the impact of comorbid serious mental illness on rehospitalization among medical and surgical inpatients. Gen Hosp Psychiatry.

[8] Abrams TE, Vaughan-Sarrazin M, Rosenthal GE (2010). Influence of psychiatric comorbidity on surgical mortality. Arch Surg.

[9] İzci Filiz, İlgün Ahmet Serkan, Fındıklı Ebru, Özmen Vahit (2016). Psychiatric Symptoms and Psychosocial Problems in Patients with Breast Cancer. J Breast Health.

[10] Montgomery GH, David D, Goldfarb AB, Silverstein JH, Weltz CR, Birk JS, Bovbjerg DH (2003). Sources of anticipatory distress among breast surgery patients. J Behav Med.

[11] Caumo W, Schmidt AP, Schneider CN, Bergmann J, Iwamoto CW, Bandeira D, Ferreira MBC (2001). Risk factors for preoperative anxiety in adults. Acta Anaesthesiol Scand.

[12] Mitchell M (2003). Patient anxiety and modern elective surgery: a literature review. J Clin Nurs.

[13] Jakobsen VH, Fagermoen MS (2005). Environmental factors in the operating theatre and their impact on patients' preoperative anxiety. Tidsskr Sykepl.

[14] Aust H, Eberhart L, Sturm T, Schuster M, Nestoriuc Y, Brehm F, Rüsch D (2018). A cross-sectional study on preoperative anxiety in adults. J Psychosom Res.

[15] Cheng JY, Wong BW, Chin YH, Ong ZH, Ng CH, Tham HY, Samarasekera DD, Devi KM, Chong CS (2021). Preoperative concerns of patients undergoing general surgery. Patient Educ Couns.

[16] Hellstadius Y, Lagergren J, Zylstra J, Gossage J, Davies A, Hultman CM, Lagergren P, Wikman A (2016). Prevalence and predictors of anxiety and depression among esophageal cancer patients prior to surgery. Dis Esophagus.

[17] Hong J, Wei Z, Wang W (2015). Preoperative psychological distress, coping and quality of life in Chinese patients with newly diagnosed gastric cancer. J Clin Nurs.

[18] Wang Y, Lu W, Shen X (2019). Assessment of preoperative psychologic distress in laryngeal cancer patients. Acta Otolaryngol.

[19] Powell R, Scott NW, Manyande A, Bruce J, Vögele C, Byrne-Davis LM, Unsworth M, Osmer C, Johnston M (2016). Psychological preparation and postoperative outcomes for adults undergoing surgery under general anaesthesia. Cochrane Database Syst Rev.

[20] Tsimopoulou I, Pasquali S, Howard R, Desai A, Gourevitch D, Tolosa I, Vohra R (2015). Psychological prehabilitation before cancer surgery: a systematic review. Ann Surg Oncol.

[21] Treanor C, Kyaw T, Donnelly M (2018). An international review and meta-analysis of prehabilitation compared to usual care for cancer patients. J Cancer Surviv.

[22] Kondylakis H, Chicchi Giglioli IA, Katehakis DG, Aldemir H, Zikas P, Papagiannakis G, Hors-Fraile S, González-Sanz PL, Apostolakis KC, Stephanidis C, Núñez-Benjumea FJ, Baños-Rivera RM, Fernandez-Luque L, Kouroubali A (2022). A digital health intervention for stress and anxiety relief in perioperative care: protocol for a feasibility randomized controlled trial. JMIR Res Protoc.

[23] Curtiss JE, Levine DS, Ander I, Baker AW (2021). Cognitive-behavioral treatments for anxiety and stress-related disorders. Focus (Am Psychiatr Publ).

[24] Ji W, Sang C, Zhang X, Zhu K, Bo L (2022). Personality, preoperative anxiety, and postoperative outcomes: a review. Int J Environ Res Public Health.

[25] Wilson CJ, Mitchelson AJ, Tzeng TH, El-Othmani MM, Saleh J, Vasdev S, LaMontagne HJ, Saleh KJ (2016). Caring for the surgically anxious patient: a review of the interventions and a guide to optimizing surgical outcomes. Am J Surg.

[26] Giraudet-Le Quintrec JS, Coste J, Vastel L, Pacault V, Jeanne L, Lamas JP, Kerboull L, Fougeray M, Conseiller C, Kahan A, Courpied JP (2003). Positive effect of patient education for hip surgery: a randomized trial. Clin Orthop Relat Res.

[27] Niknejad R, Mirmohammad-Sadeghi M, Akbari M, Ghadami A (2019). Effects of an orientation tour on preoperative anxiety in candidates for coronary artery bypass grafting: a randomized clinical trial. ARYA Atheroscler.

[28] Haugen AS, Eide GE, Olsen MV, Haukeland B, Remme ÅR, Wahl AK (2009). Anxiety in the operating theatre: a study of frequency and environmental impact in patients having local, plexus or regional anaesthesia. J Clin Nurs.

[29] Musa A, Movahedi R, Wang JC, Safani D, Cooke C, Hussain SF, Tajran J, Hamid S, Gucev G (2020). Assessing and reducing preoperative anxiety in adult patients: a cross-sectional study of 3661 members of the American Society of Anesthesiologists. J Clin Anesth.

[30] Harbell MW, Dumitrascu C, Bettini L, Yu S, Thiele CM, Koyyalamudi V (2021). Anesthetic considerations for patients on psychotropic drug therapies. Neurol Int.

[31] Maurice-Szamburski A, Auquier P, Viarre-Oreal V, Cuvillon P, Carles M, Ripart J, Honore S, Triglia T, Loundou A, Leone M, Bruder N, PremedX Study Investigators (2015). Effect of sedative premedication on patient experience after general anesthesia: a randomized clinical trial. JAMA.

[32] Parsons TD (2015). Virtual reality for enhanced ecological validity and experimental control in the clinical, affective and social neurosciences. Front Hum Neurosci.

[33] Rizzo A, Buckwalter JG, John B, Newman B, Parsons T, Kenny P, Williams J (2012). STRIVE: stress resilience in virtual environments: a pre-deployment VR system for training emotional coping skills and assessing chronic and acute stress responses. Stud Health Technol Inform.

[34] Ilnicki S, Wiederhold BK, Maciolek J, Kosinska L, Szymanska S, Zbyszewski M, Siatkowska A, Opalko-Piotrkiewicz E, Ilnicki P, Filarowska M, Glibowska A, Borzetka D, Pleskacz K, Murawski P (2012). Effectiveness evaluation for short-term group pre-deployment VR computer-assisted stress inoculation training provided to Polish ISAF soldiers. Stud Health Technol Inform.

[35] Stanley EA, Schaldach JM, Kiyonaga A, Jha AP (2011). Mindfulness-based mind fitness training: a case study of a high-stress predeployment military cohort. Cogn Behav Pract.

[36] Rothbaum BO, Hodges LF, Ready D, Graap K, Alarcon RD (2001). Virtual reality exposure therapy for Vietnam veterans with posttraumatic stress disorder. J Clin Psychiatry.

[37] Bekelis K, Calnan D, Simmons N, MacKenzie TA, Kakoulides G (2017). Effect of an immersive preoperative virtual reality experience on patient reported outcomes: a randomized controlled trial. Ann Surg.

[38] Ryu JH, Park SJ, Park JW, Kim JW, Yoo HJ, Kim TW, Hong JS, Han SH (2017). Randomized clinical trial of immersive virtual reality tour of the operating theatre in children before anaesthesia. Br J Surg.

[39] Gold JI, Annick ET, Lane AS, Ho K, Marty RT, Espinoza JC (2021). "Doc McStuffins: doctor for a day" virtual reality (DocVR) for pediatric preoperative anxiety and satisfaction: pediatric medical technology feasibility study. J Med Internet Res.

[40] Yu Y, Zhou X, Zeng G, Hou Y (2023). Impact of virtual operating room tours on relieving perioperative anxiety in adult patients: a systematic review. J Perianesth Nurs.

[41] Mbewe AM, Smith Z (2023). Effectiveness of virtual reality interventions to reduce pre-operative anxiety in adult surgical patients in the pre-operative period: systematic review and meta-analysis. J Perioper Nurs.

[42] Turrado V, Guzmán Y, Jiménez-Lillo J, Villegas E, de Lacy FB, Blanch J, Balibrea JM, Lacy A (2021). Exposure to virtual reality as a tool to reduce peri-operative anxiety in patients undergoing colorectal cancer surgery: a single-center prospective randomized clinical trial. Surg Endosc.

[43] Vogt L, Klasen M, Rossaint R, Goeretz U, Ebus P, Sopka S (2021). Virtual reality tour to reduce perioperative anxiety in an operating setting before anesthesia: randomized clinical trial. J Med Internet Res.

[44] Noben L, Goossens SM, Truijens SE, van Berckel MM, Perquin CW, Slooter GD, van Rooijen SJ (2019). A virtual reality video to improve information provision and reduce anxiety before cesarean delivery: randomized controlled trial. JMIR Ment Health.

[45] Morgan H, Nana M, Phillips D, Gallagher S (2021). The effect of a VIrtual RealiTy immersive experience upon anxiety levels, procedural understanding, and satisfaction in patients undergoing CArdiac CaTHeterization: the Virtual Cath trial. J Invasive Cardiol.

[46] Kapikiran G, Bulbuloglu S, Saritas S (2022). The effect of video training before organ transplant surgery on patient satisfaction and anxiety: head mounted display effect. Clin Simul Nurs.

[47] Keshvari M, Yeganeh MR, Paryad E, Roushan ZA, Pouralizadeh M (2021). The effect of virtual reality distraction on reducing patients' anxiety before coronary angiography: a randomized clinical trial study. Egypt Heart J.

[48] Robertson A, Khan R, Fick D, Robertson WB, Gunaratne DR, Yapa S, Bowden V, Hoffman H, Rajan R (2017). The effect of virtual reality in reducing preoperative anxiety in patients prior to arthroscopic knee surgery: a randomised controlled trial. Proceedings of the 5th International Conference on Serious Games and Applications for Health.

[49] Hendricks TM, Gutierrez CN, Stulak JM, Dearani JA, Miller JD (2020). The use of virtual reality to reduce preoperative anxiety in first-time sternotomy patients: a randomized controlled pilot trial. Mayo Clin Proc.

[50] Ionescu A, Van Daele T, Rizzo A, Blair C, Best P (2021). 360° videos for immersive mental health interventions: a systematic review. J Technol Behav Sci.

[51] Petersen GB, Petkakis G, Makransky G (2022). A study of how immersion and interactivity drive VR learning. Comput Educ.

[52] Donnelly MR, Reinberg R, Ito KL, Saldana D, Neureither M, Schmiesing A, Jahng E, Liew SL (2021). Virtual reality for the treatment of anxiety disorders: a scoping review. Am J Occup Ther.

[53] Maples-Keller JL, Bunnell BE, Kim SJ, Rothbaum BO (2017). The use of virtual reality technology in the treatment of anxiety and other psychiatric disorders. Harv Rev Psychiatry.

[54] Martens MA, Antley A, Freeman D, Slater M, Harrison PJ, Tunbridge EM (2019). It feels real: physiological responses to a stressful virtual reality environment and its impact on working memory. J Psychopharmacol.

[55] Birckhead B, Khalil C, Liu X, Conovitz S, Rizzo A, Danovitch I, Bullock K, Spiegel B (2019). Recommendations for methodology of virtual reality clinical trials in health care by an international working group: iterative study. JMIR Ment Health.

[56] (2024). National research council Canada. Government of Canada.

[57] Sommer JL, Reynolds K, Hebbard P, Smith MS, Mota N, Mutch WA, Maples-Keller J, Roos L, El-Gabalawy R (2020). Preoperative virtual reality for cancer surgery patients: a feasibility and pilot study. ClinicalTrials.

[58] Blatch-Jones AJ, Pek W, Kirkpatrick E, Ashton-Key M (2018). Role of feasibility and pilot studies in randomised controlled trials: a cross-sectional study. BMJ Open.

[59] Cocks K, Torgerson DJ (2013). Sample size calculations for pilot randomized trials: a confidence interval approach. J Clin Epidemiol.

[60] Haahr M True random number service. RANDOM.ORG.

[61] Crockett JK, Gumley A, Longmate A (2007). The development and validation of the Pre-operative Intrusive Thoughts Inventory (PITI). Anaesthesia.

[62] Moerman N, van Dam FS, Muller MJ, Oosting H (1996). The Amsterdam Preoperative Anxiety and Information Scale (APAIS). Anesth Analg.

[63] Tang L, Zhang Y, Pang Y, Zhang H, Song L (2011). Validation and reliability of distress thermometer in chinese cancer patients. Chin J Cancer Res.

[64] Graham-Wisener L, Dempster M, Sadler A, McCann L, McCorry NK (2021). Validation of the distress thermometer in patients with advanced cancer receiving specialist palliative care in a hospice setting. Palliat Med.

[65] Brunet A, Weiss DS, Metzler TJ, Best SR, Neylan TC, Rogers C, Fagan J, Marmar CR (2001). The Peritraumatic Distress Inventory: a proposed measure of PTSD criterion A2. Am J Psychiatry.

[66] Prins A, Bovin MJ, Smolenski DJ, Marx BP, Kimerling R, Jenkins-Guarnieri MA, Kaloupek DG, Schnurr PP, Kaiser AP, Leyva YE, Tiet QQ (2016). The Primary Care PTSD Screen for DSM-5 (PC-PTSD-5): development and evaluation within a veteran primary care sample. J Gen Intern Med.

[67] Sinclair VG, Wallston KA (2004). The development and psychometric evaluation of the Brief Resilient Coping Scale. Assessment.

[68] Schalet BD, Pilkonis PA, Yu L, Dodds N, Johnston KL, Yount S, Riley W, Cella D (2016). Clinical validity of PROMIS® depression, anxiety, and anger across diverse clinical samples. J Clin Epidemiol.

[69] Park J, Rodriguez JL, O'Brien KM, Nichols HB, Hodgson ME, Weinberg CR, Sandler DP (2021). Health-related quality of life outcomes among breast cancer survivors. Cancer.

[70] Cella D, Lai J, Jensen SE, Christodoulou C, Junghaenel DU, Reeve BB, Stone AA (2016). PROMIS fatigue item bank had clinical validity across diverse chronic conditions. J Clin Epidemiol.

[71] Hahn EA, Cella D, Bode RK, Hanrahan RT (2009). Measuring social well-being in people with chronic illness. Soc Indic Res.

[72] Grocott B, Reynolds K, Logan G, Hebbard P, El-Gabalawy R (2023). Breast cancer patient experiences of perioperative distress and anxiety: a qualitative study. Eur J Oncol Nurs.

[73] Schubert TW (2003). The sense of presence in virtual environments: a three-component scale measuring spatial presence, involvement, and realness. J Media Psychol.

[74] Braun V, Clarke V (2006). Using thematic analysis in psychology. Qual Res Psychol.

[75] Braun V, Clarke V (2019). Reflecting on reflexive thematic analysis. Qual Res Sport Exerc Health.

[76] Braun V, Clarke V (2023). Is thematic analysis used well in health psychology? A critical review of published research, with recommendations for quality practice and reporting. Health Psychol Rev.

[77] Sommer JL, Reynolds K, Hebbard P, Smith MS, Mota N, Mutch WA, Maples-Keller J, Roos L, El-Gabalawy R (2024). Preoperative virtual reality to expose patients with breast cancer to the operating room environment: feasibility and pilot case series study. JMIR Form Res.

[78] Dhar E, Upadhyay U, Huang Y, Uddin M, Manias G, Kyriazis D, Wajid U, AlShawaf H, Syed Abdul S (2023). A scoping review to assess the effects of virtual reality in medical education and clinical care. Digit Health.

[79] Montgomery GH, Bovbjerg DH (2004). Presurgery distress and specific response expectancies predict postsurgery outcomes in surgery patients confronting breast cancer. Health Psychol.

[80] Montgomery GH, Schnur JB, Erblich J, Diefenbach MA, Bovbjerg DH (2010). Presurgery psychological factors predict pain, nausea, and fatigue one week after breast cancer surgery. J Pain Symptom Manage.

