# Supplementary material for: An Immersive Virtual Reality Intervention for Preoperative Anxiety and Distress Among Adults Undergoing Oncological Surgery: Protocol for a 3-Phase Development and Feasibility Trial
Source: JMIR Res Protoc. 2024 May 14;13:e55692. doi: 10.2196/55692 (PMC11134251; doi:10.2196/55692)
Supplement: Multimedia Appendix 1 [file resprot_v13i1e55692_app1.docx]

Appendix 1. Timeline of ethics submission for the VR feasibility study, with dates and description of reason for submission

| **Date of ethics submission** | **Type of ethics submission with brief description** |
| --- | --- |
| **May 1, 2020** | **New ethics submission**  - Submitted original protocol for VR OR study with outcome measures and recruitment methods |
| **Jan 24, 2021** | **Amendment submission**  -Added Brief Resilient Coping Scale  -Added standardized observation form |
| **Sept 8, 2021** | **Amendment submission**  -Adding a UM institutionally required consent form for participants regarding the risk of in-person research during the COVID-19 pandemic, informing them of safety precautions undertaken by research personnel  -Clearance of medical personnel passing patient information to research personnel |
| **April 26, 2022** | **Amendment submission**  -Broadened sample to include other cancer surgery types  -Dropped VR control group due to slow recruitment  -Added possibility of home-visits for VR intervention |
| **Nov 4, 2022** | **Amendment submission**  -Added case series analysis and verbal consent from participants to allow for this interim analysis  -Added ability to break randomization if needed |
| **June 5, 2023** | **New ethics submission**  -Submitted new linked ethics application to allow for contact of past participants recruitment for Phase 3 focus groups |
